# Commentary: Precision Immunotherapy for Sepsis

**DOI:** 10.3389/fimmu.2019.00020

**Published:** 2019-01-31

**Authors:** Jan G. Zijlstra, Matijs van Meurs, Jill Moser

**Affiliations:** Department of Critical Care, University of Groningen, University Medical Center Groningen, Groningen, Netherlands

**Keywords:** sepsis, immunotherapy, immunosupression, immunoparalysis, endotypes, hyperinflammtion

We read with interest the review article published in *Frontiers in Immunology* by Peters van Ton et al., who suggest that precision immunotherapy might benefit organ failure and reduce the mortality of sepsis patients. Sepsis is still an enormous health-threat worldwide and mortality rates are still very high despite recent advances in early recognition. In that sense, we agree with Peters van Ton and colleagues, a therapy that will reduce mortality and morbidity in sepsis patients is urgently needed. The question is; will that be immunotherapy?

More than a century ago bacterial infection was recognized as the cause of sepsis. When antibiotics did not save all patients, the logical step was to suspect a failing immune system. The immune system is clearly an early and active responder during organ failure in sepsis but there is more to the host response than just a derailed immune system. What clinical evidence really supports the immune system as the key “organ” causing death in sepsis patients?

As the authors state, decades of failed clinical trials focusing on immunomodulating therapies have not resulted in a new therapy for sepsis. Back in 1996, Roger Bone discussed the first failed clinical trials and already suggested then, that the model of persistent, uncontrolled inflammation was inaccurate (1). He pleaded that we should learn from our mistakes and emphasized the necessity to examine all of the physiological responses the body was capable of mounting, not simply the most or least severe (1). Instead, his advice was largely ignored, and a further 25 years of clinical trials targeting the hyperinflammatory response in sepsis patients ensued; primarily driven by the hope of finding the “magic bullet,” but also fueled by the pharmaceutical industry. At that time, Bone mentioned that the anti-inflammatory response had, for the most part, been ignored. However, in recent years it has gained increasing attention mainly due to the many clinical trials that were unable to improve patient outcome. Sepsis induced-“immunosuppression” or “immunoparalysis” is now thought to be one of the main drivers of mortality and morbidity in patients, which has led to the birth of immunostimulatory compounds as a potential new therapy.

However, we fear that switching from taming an overactive immune system to stimulating a depressed system, or even regulating the immune system on-demand will end up in new disappointments. Moreover, recent evidence suggests that it's not *either-or*, patients can become hyperinflammed and immunosuppressed concurrently (2), questioning the usefulness and safety of immunostimulatory compounds. We therefore agree with the authors, identifying patients that might benefit from immunostimulatory compounds and those that might not is extremely crucial.

Many believe that patients succumb to sepsis as a result of secondary infection due to immunosuppression and the inability to fight infection. Although this might be the case for some patients, it certainly doesn't hold true for all patients with sepsis. Van Vught et al., showed in their study that only 13.5% of all sepsis ICU-admissions developed secondary ICU-acquired infection. Despite these patients having a higher disease severity score at admission the contribution of secondary infection on overall mortality was low (3). The authors also refer to studies showing that pre-exposure to bacterial products resulted in improved clearance and survival upon rechallenge with live bacteria (4, 5). Moreover, a causal relationship between immune suppression and mortality from ICU-acquired secondary infection has not yet been reported.

The authors also highlight an important concept, it is currently unknown whether the immune status of organs in septic patients is comparable to the immune status within the blood compartment. This knowledge is clinically important, since as the authors state, tissue resident macrophages and other cells appear to be primarily responsible for the innate immune response in sepsis rather than the circulating immune cells. Whether treatment with immunostimulatory compounds results in further organ dysfunction is also currently unknown. One might imagine that treatment with immunostimulatory compounds that promote systemic TNFα release within the blood compartment of immunosuppressed patients may be beneficial if they need to fight secondary infection (6, 7), but it may also result in detrimental cellular responses resulting in further organ function deterioration.

Expecting immunotherapy to diminish organ failure in all patients with sepsis is unrealistic. Recent studies have identified sepsis endotypes with different clinical and molecular profiles (8, 9). The host-response to sepsis therefore differs per patient implying the need for different treatment strategies rather than the traditional “one-size-fits-all” approach. These types of studies should be embraced since this will unquestionably promote recognition of specific groups for precise therapy as well as advancing better clinical trial design.

There is also a danger that the immunotherapy hype will overshadow investment into other promising therapies targeting other aspects of organ failure. This would be unfortunate since some patients will benefit from immunotherapy whereas others may benefit from a different type of treatment, or more likely, a combination of different therapies. In that sense, we really have to broaden our view and support translational research aimed at further elucidating the other “host-responses” and mechanisms that may be mediating organ failure in patients with sepsis, in order to uncover treatment strategies other than immunotherapy.

## Author Contributions

JZ and JM: conceived, drafted, and wrote this commentary; MvM provided important intellectual contribution. All authors read and approved the submitted version.

### Conflict of Interest Statement

The authors declare that the research was conducted in the absence of any commercial or financial relationships that could be construed as a potential conflict of interest.

## References

[1] Bone RC. Sir Isaac Newton, sepsis, SIRS, and CARS. Crit Care Med. (1996) 24:1125–8. 8674323 10.1097/00003246-199607000-00010

[2] van Vught LA Wiewel MA Hoogendijk AJ Frencken JF Scicluna BP Klein Klouwenberg PMC. The host response in patients with sepsis developing intensive care unit-acquired secondary infections. Am J Respir Crit Care Med. (2017) 196:458–70. 10.1164/rccm.201606-1225OC 28107024

[3] van Vught LA Klein Klouwenberg PM Spitoni C Scicluna BP Wiewel MA Horn J. Incidence, risk factors, and attributable mortality of secondary infections in the intensive care unit after admission for sepsis. JAMA (2016) 315:1469–79. 10.1001/jama.2016.2691 26975785

[4] Murphey ED Fang G Sherwood ER. Endotoxin pretreatment improves bacterial clearance and decreases mortality in mice challenged with *Staphylococcus aureus*. Shock (2008) 29:512–8. 10.1097/shk.0b013e318150776f 17724430

[5] Murphey ED Sherwood ER. Pretreatment with the Gram-positive bacterial cell wall molecule peptidoglycan improves bacterial clearance and decreases inflammation and mortality in mice challenged with *Pseudomonas aeruginosa*. Microbes Infect. (2008) 10:1244–50. 10.1016/j.micinf.2008.07.021 18678270 PMC2603308

[6] Leentjens J Kox M Koch RM Preijers F Joosten LA van der Hoeven JG. Reversal of immunoparalysis in humans *in vivo*: a double-blind, placebo-controlled, randomized pilot study. Am J Resp Crit Care Med. (2012) 186:838–45. 10.1164/rccm.201204-0645OC 22822024

[7] Zhang Y Zhou Y Lou J Li J Bo L Zhu K. PD-L1 blockade improves survival in experimental sepsis by inhibiting lymphocyte apoptosis and reversing monocyte dysfunction. Crit Care (2010) 14:R220. 10.1186/cc9354 21118528 PMC3220038

[8] Famous KR Delucchi K Ware LB Kangelaris KN Liu KD Thompson BT. Acute respiratory distress syndrome subphenotypes respond differently to randomized fluid management strategy. Am J Respir Crit Care Med. (2017) 195:331–8. 10.1164/rccm.201603-0645OC 27513822 PMC5328179

[9] Sweeney TE Azad TD Donato M Haynes WA Perumal TM Henao R. Unsupervised analysis of transcriptomics in bacterial sepsis across multiple datasets reveals three robust clusters. Crit Care Med. (2018) 46:915–25. 10.1097/CCM.0000000000003084 29537985 PMC5953807

